# Deep Learning-Based Defects Detection in Keyhole TIG Welding with Enhanced Vision

**DOI:** 10.3390/ma17153871

**Published:** 2024-08-05

**Authors:** Xuan Zhang, Shengbin Zhao, Mingdi Wang

**Affiliations:** School of Mechanical and Electrical Engineering, Soochow University, Suzhou 215137, China; 20224229033@stu.suda.edu.cn

**Keywords:** keyhole TIG welding, deep learning, defects detection

## Abstract

Keyhole tungsten inert gas (keyhole TIG) welding is renowned for its advanced efficiency, necessitating a real-time defect detection method that integrates deep learning and enhanced vision techniques. This study employs a multi-layer deep neural network trained on an extensive welding image dataset. Neural networks can capture complex nonlinear relationships through multi-layer transformations without manual feature selection. Conversely, the nonlinear modeling ability of support vector machines (SVM) is limited by manually selected kernel functions and parameters, resulting in poor performance for recognizing burn-through and good welds images. SVMs handle only lower-level features such as porosity and excel only in detecting simple edges and shapes. However, neural networks excel in processing deep feature maps of “molten pools” and can encode deep defects that are often confused in keyhole TIG. Applying a four-class classification task to weld pool images, the neural network adeptly distinguishes various weld states, including good welds, burn-through, partial penetration, and undercut. Experimental results demonstrate high accuracy and real-time performance. A comprehensive dataset, prepared through meticulous preprocessing and augmentation, ensures reliable results. This method provides an effective solution for quality control and defect prevention in keyhole TIG welding process.

## 1. Introduction

The evolving landscape of the manufacturing industry underscores the significance of keyhole TIG welding technology in industrial operations [1]. As industries increasingly demand higher precision and efficiency, advanced welding techniques like keyhole TIG are crucial. However, the occurrence of flaws during keyhole TIG welding processes can diminish product efficacy and pose safety risks [2]. Consequently, the prompt and precise identification and evaluation of keyhole TIG welding imperfections have emerged as pivotal areas of research in this welding technique. Wang Z and collaborators are presently engaged in a study investigating the operational dynamics of keyhole TIG welding [3], with the objective of crafting a monitoring system customized for contemporary industries. This initiative endeavors to uphold production standards and elevate automation levels in manufacturing processes.

In academic literature, the utilization of an infrared thermal imager has been documented for defect detection in keyhole TIG cladding [4]. This technology facilitates non-contact scanning of the cladding area, mitigating the risk of potential surface damage. Moreover, the infrared thermal imager possesses the capability to penetrate certain material thicknesses, aiding in the detection of internal defects [5]. However, it is essential to recognize that thermal infrared cameras have inherent limitations in resolution, rendering them unable to discern small defects effectively. The processing of the temperature data collected necessitates intricate procedures for defect identification [6]. In contrast, images generated through deep learning exhibit an enhanced capacity to capture more detailed feature information and demonstrate increased resilience against environmental interferences. Deep learning images can effectively capture intricate features, thereby augmenting recognition accuracy [7,8].

Keyhole TIG welding visual inspection utilizes tools like TensorFlow to extract spatial features for real-time automatic defect detection. In contrast, spectral detection lacks spatial richness and necessitates complex algorithms, high hardware standards, including expensive equipment, and periodic calibration. High-speed cameras offer a more economical solution and facilitate the acquisition of high-quality visual data. Traditional defect detection often faces challenges in achieving the necessary precision and real-time capability, primarily due to the intricate and varied characteristics of welding images. This study aims to address this issue by utilizing deep learning algorithms and vision techniques for defect detection in welding processes. A defect recognition strategy based on the residual neural network architecture has been formulated. By analyzing four categories of molten pool images, the model adeptly monitors each stage of the welding process.

Compared with the references [9,10], this study introduces multi-layer deep neural networks that can adaptively extract complex nonlinear features instead of relying on the manual selection of features and kernel functions. In addition, by using the optimized K-SVD algorithm for image preprocessing, the image quality and the generalization ability of the model are significantly improved, which ensures reliability in practical industrial applications.

To extract intricate features from welding visual data, a sophisticated deep network structure has been devised, incorporating convolutional layers, spatial pooling layers, and fully connected layers. The convolutional layer extracts detailed features through filters [11], the spatial pooling layer reduces the image’s spatial dimensions [12], and the fully connected layer conducts comprehensive analysis and renders classification decisions.

A series of experiments were conducted to demonstrate the effectiveness of the proposed method. Different welding parameters, such as current, voltage, and travel speed, and various types of defects, including porosity, cracks, and incomplete fusion, were defined and studied through experimentation. Welding tests were performed on materials of different grades, including stainless steel, aluminum alloys, and titanium alloys, to cover a broad range of industrial applications. Through the collection of welding images and subsequent data preprocessing and enhancement, a dependable dataset was assembled. The experimental findings validate the efficiency and accuracy of the proposed deep learning technology in identifying defects in keyhole TIG welding.

## 2. Methods

### 2.1. Experimental Setup

**Figure 1:**
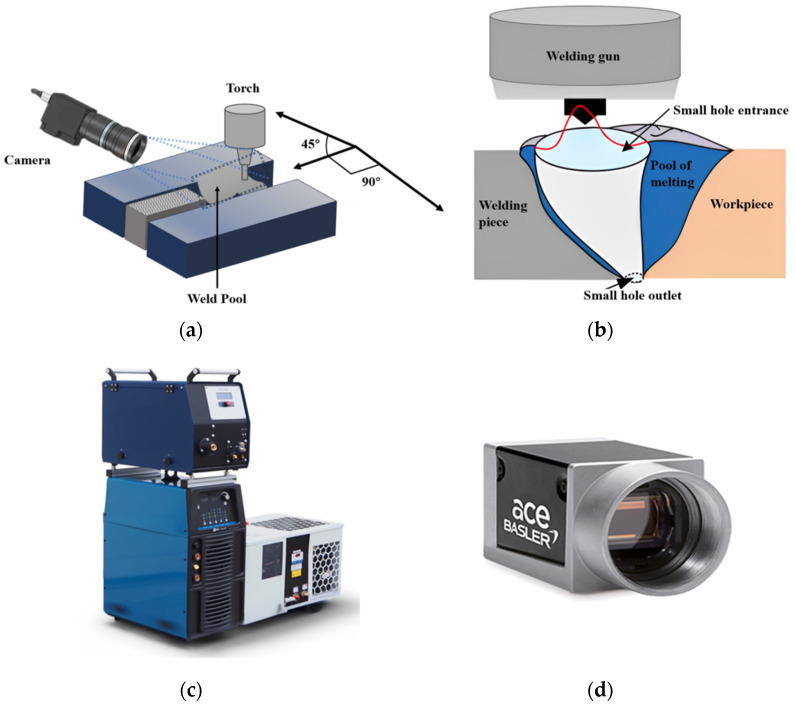
Schematic (**a**) TIG welding process layout, (**b**) Welding diagram, (**c**) TIG welding machine, (**d**) Camera.

The study entailed conducting experiments on a 304 stainless steel specimen measuring 250 mm in length, 150 mm in width, and 10 mm in thickness. Tungsten inert gas (TIG) welding was executed employing pure argon as the shielding gas at a flow rate of 30 L per minute. The welding process encompassed a travel speed of 20 cm per minute and an operating voltage of 20 volts. A 6.5 mm diameter tungsten electrode with a minor lanthanum addition was utilized to fulfill the electrical prerequisites for TIG welding, with the distance between the tungsten tip and the workpiece set at 2.5 mm. For monitoring purposes, a camera was mounted on a robotic arm to track the weld seam, positioned at a 45° angle relative to the welding direction, as depicted in Figure 1. This orientation was selected to facilitate optimal visualization of the region directly in front of the welding pool and arc.

In Figure 1b, the red line represents the arc path in the welding process. TIP TIG’s KTIG equipment is produced by the German manufacturer TIP TIG GmbH and is headquartered in Alsdorf, Germany. The Basler ace acA1920-40gm camera is manufactured by Basler AG, headquartered in Ahrensburg, Germany. Both devices are available worldwide.

### 2.2. Experiment Process

The welding process involves using a non-consumable tungsten electrode to produce the weld. The welding area is protected from atmospheric contamination by an inert shielding gas, usually argon. In our study, we experimented with 304 stainless steel specimens using a travel speed of 20 cm/min and an operating voltage of 20 volts. 304 stainless steel sheets were chosen due to their widespread industrial use and well-documented welding characteristics, making them ideal for validating the effectiveness of the TIG welding process and defect detection model.

**Figure 2:**
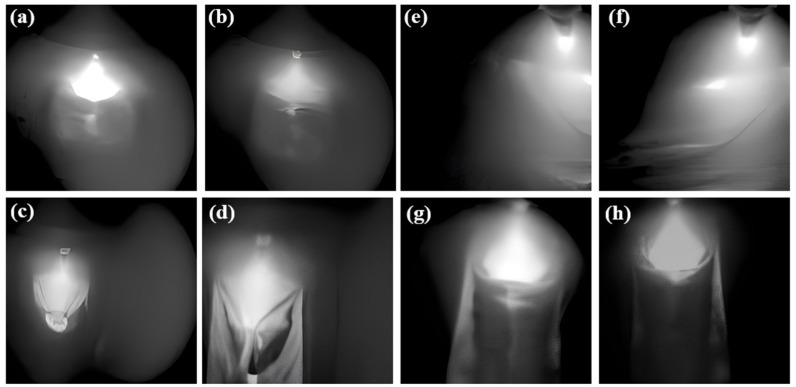
Samples under different labels of the dataset in the molten pool, (**a**,**b**) Good weld, (**c**,**d**) Burn through (**e**,**f**) partial-penetration, (**g**,**h**) Undercut.

In this research, four distinct welding states were delineated to attain diverse welding outcomes. These states encompass good weld, burn through, partial-penetration, and undercut. The welding process parameters and their corresponding welding states are delineated in Table 1. Each welding state was scrutinized through four distinct experiments, each characterized by a unique welding current setting. A total of 1000 images were captured for each experiment. Figure 2 illustrates the external appearance of the molten pool under varying welding conditions. It is evident that each welding condition yields a distinctive molten pool structure. Under optimal conditions, the keyhole and molten pool exhibit a relatively uniform morphology. Conversely, during the burn through state, a distinct circular or elliptical penetration hole is observable at the weld seam, offering a direct view of the underlying surface. Surrounding the penetration hole are remnants of splattered molten metal.

**Figure 3:**
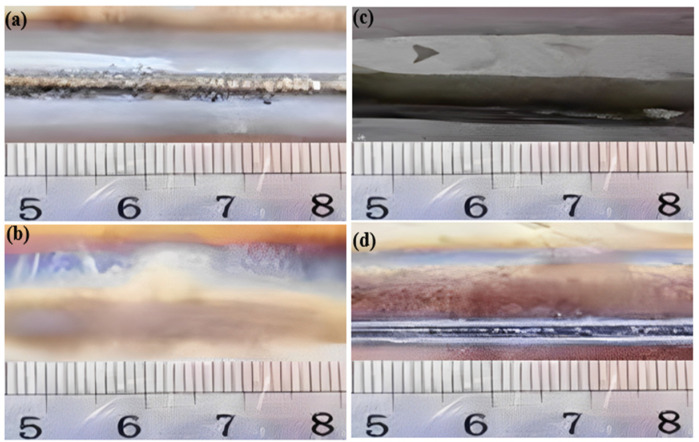
Illustrations of weld bead appearances, (**a**) Good weld, (**b**) Burn through, (**c**) Partial penetration, (**d**) Undercut.

Insufficient heat input during welding, typically with a current ranging from 330 to 350 amperes, may result in the occurrence of partial penetration. This phenomenon is characterized by a visible weld bead line in the unfused area, exhibiting discontinuity and an uneven width of the weld bead. Figure 2 and Figure 3 depict small gaps or lines within specific sections of the weld bead, indicative of inadequate fusion between the material layers. Furthermore, Figure 3 illustrates undercut as V-shaped indentations situated at the edges of the weld seam. Irregularities in depth along the edges of the weld seam are noticeable, with discernible indentations present. These indentations have the potential to impact both the structural integrity and visual appeal of the weld seam.

### 2.3. Machine Learning-Based Image Processing

**Figure 4:**
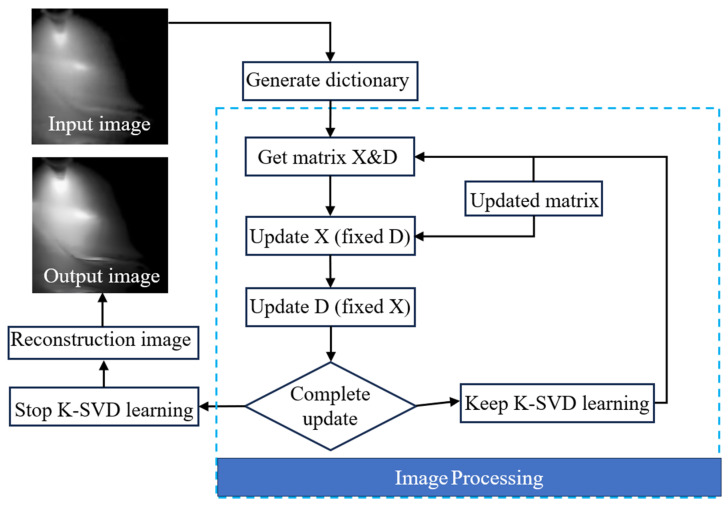
Flow chart of the K-SVD algorithm.

In the realm of deep learning, particularly in the advanced application of convolutional neural networks (CNNs), the precise enhancement of welding images holds significant importance [13]. The efficacy of these models hinges heavily on the quality and integrity of the input data, emphasizing the need for refining preprocessing methods as a critical strategic choice. In comparison to traditional welding methods, keyhole TIG welding generates a more intense arc light during the welding process [14]. Despite efforts to mitigate arc light through the use of optical filters, this approach often results in the production of blurred welding images and reduced brightness in areas distant from the arc’s center [15,16]. This diminished visibility can compromise the characteristics of the images and impede the accurate extraction of features by deep learning networks. Therefore, the K-SVD algorithm is employed to enhance welding images by leveraging nonlinear spatial transformations, color constancy, and local contrast enhancement. This algorithm adeptly distinguishes between illumination and reflection in images, rectifies uneven illumination, and enhances contrast and detail information. The study is centered on utilizing the K-SVD algorithm to dehaze and enhance keyhole TIG welding images. Moreover, optimization strategies are devised to formulate a more resilient learning structure that mitigates overfitting tendencies, thereby enhancing the model’s capacity to generalize to new datasets. The specific procedures for image optimization in the experiments are delineated in Figure 4.

Singular value decomposition (SVD) is applied to the images, decomposing the image matrix into three matrices: *U*, *Σ*, and the transpose of *V*, as illustrated in Equation (1).

(1) $$A=U\sum V^{T}$$

**Figure 5:**
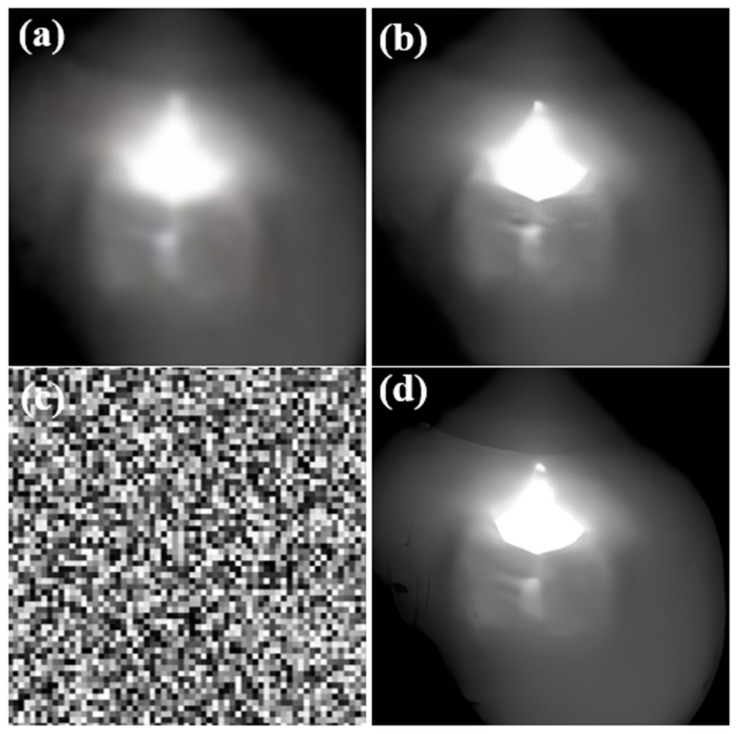
Defect imaging enhancement process, (**a**) Original image, (**b**) Gaussian-filtered, (**c**) Dictionary matrix in K-SVD algorithm, (**d**) Final imaging.

The primary energy components of an image are encapsulated in a diagonal matrix denoted as *Σ*. By truncating *Σ* and retaining only the top 5 singular values, the image’s dimensionality can be reduced while preserving its key characteristics, resulting in denoising and compression effects. The image data processed through singular value decomposition (SVD) is subsequently subjected to analysis using the K-Means clustering algorithm on the preprocessed images. This algorithm classifies the pixels in the image into four distinct defect groups, each representing a significant material area. The optimization process for images is depicted in Figure 5, illustrating that the application of SVD effectively eliminates molten material from the image, thereby enhancing the clarity of details in the molten pool region.

### 2.4. Dataset

The research focused on investigating various welding conditions both with and without common defects encountered during welding processes. Video recordings were conducted at a rate of 45 frames per second, utilizing an original image resolution of 1280 × 1024 pixels. The camera employed in the experiments boasted a dynamic range exceeding 140 dB, enabling it to effectively capture light and enhance brightness around the welding arc without inducing excessive expansion of the arc.

Upon completion of model training, a cross-testing set was established to replicate the model’s performance in identifying various types of TIG welding defects in real-world scenarios. It was imperative to validate the effectiveness of unfamiliar data by ensuring that the testing set data was distinct from the TIG welding image data used in the training and cross-validation sets.

In the development of the algorithm model, a partitioning ratio of 6:2:2 was applied for the training set, testing set, and validation set. This distribution was chosen to minimize interdependencies among subsets and evaluate the network’s capability to adapt to and represent defects, as indicated in previous studies. The dataset composition utilized in the research is outlined in Table 2. The training set for defect images constituted 60% of the total image recognition dataset, comprising 4000 images. This partitioning approach facilitated the enhancement of image defect identification algorithms in terms of accuracy, resilience, and dependability. The images captured during the experiments were focused on capturing the areas surrounding the molten pool and the welding arc.

To prevent overfitting in our defect detection model, we applied data augmentation, dropout, cross-validation, and early stopping. These measures enhanced the model’s generalizability, ensuring reliable defect detection across various welding conditions. Our approach focused on capturing molten pool images to train a robust deep learning model.

### 2.5. ResNet Model

The residual network (ResNet) represents a significant breakthrough in addressing the challenge of the “degradation problem” encountered during the training of deep neural networks [17]. This issue is particularly prevalent in complex models, such as deep neural networks, where training errors may not consistently decrease as the number of layers increases. This phenomenon is often attributed to the vanishing and exploding gradients problem [18], which becomes more pronounced in extremely deep networks. The ResNet model introduces a novel approach by incorporating a residual learning framework, setting it apart from conventional networks [19]. This framework focuses on learning the difference between the input and the desired output, simplifying the learning process [20]. As a result, the network can effectively skip redundant predictions as it integrates more layers. This approach helps alleviate the performance degradation that can occur with increased depth, ensuring the feasibility of highly intricate networks [21,22]. Consequently, this strategy significantly enhances training efficiency and boosts the model’s effectiveness.

In advanced deep learning research, the primary strengths of ResNet can be categorized into three main advantages [23]. Firstly, it enhances the robustness of deep network training, enabling models to extract intricate image features more effectively [24]. Secondly, it enhances the optimization of gradient backpropagation, thereby addressing the fundamental issue of gradient vanishing. Thirdly, it expands the potential applications and complexity of models in tasks related to complex visual understanding [25], particularly in fields such as high-resolution image segmentation, deep semantic feature-dependent image retrieval systems, and other areas that have witnessed significant advancements [26].

**Figure 6:**
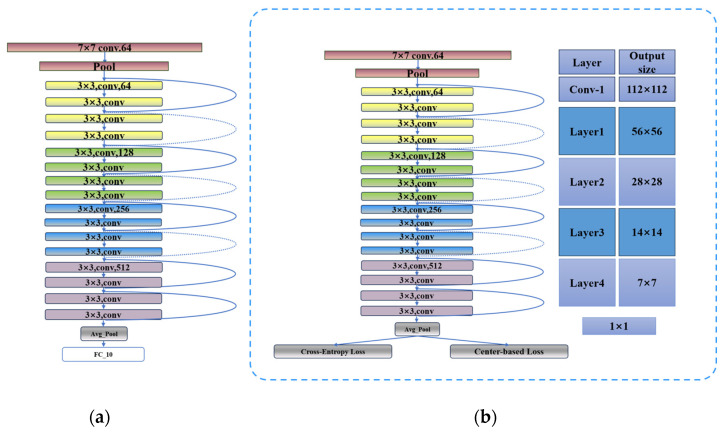
(**a**) Architecture of the traditional ResNet and (**b**) architecture of the improved ResNet. Different colors correspond to different layers.

In this study, ResNet with an 18-layer convolutional architecture served as the foundational model. To enhance the model training process, a metric learning technique employing the attention mechanism was introduced. A unique two-stream parallel training method was implemented, which innovatively incorporated the central loss mechanism while preserving the traditional softmax loss function to maintain class discrimination ability. The specific structural arrangement is illustrated in Figure 6, where the center loss component is intricately connected to the output terminal of the global average pooling layer. During the backpropagation phase, the system can simultaneously manage gradient signals from both the softmax loss and center loss, enabling a dual-path gradient feedback approach. In Figure 6, different colors correspond to different layers.

Traditional softmax loss indeed excels in multi-class discrimination [27], yet its capacity to discern subtle differences within images is somewhat limited. It is within this context that the integration of metric learning principles into network design strategies becomes particularly pivotal. In the case of the center loss algorithm, its distinctiveness lies in dynamically adjusting the learning path based on the Euclidean distance deviation between samples and their class centers. Furthermore, these class centers continuously optimize and update throughout the training process, ensuring high precision and adaptability in model learning. The mathematical formulation of this process is elaborated in Equation (2).

(2) $$L_{\mathrm{center}}=\frac{1}{2}\sum_{i=1}^{N}\|f(x_{i})-c_{yi}{\|}_{2}^{2}$$

In Equation (3), *f(x_i_)* represents the deep features in the embedding space, *C_y_*_i_ denotes the center of the *y_i_-th* class, and N represents the batch size. The total loss of the network is considered as a combination of softmax loss and center loss.

(3) $$Loss=L_{cls}+\lambda Lcenter$$

In the equation, *L_cls_* represents the softmax loss, and *λ* is the weight of the center loss. In this study, *λ* is set to 1, which is a commonly used and beneficial value in multi-classification tasks [25,26].

## 3. Results and Discussion

### 3.1. Model Processing Results

**Figure 7:**
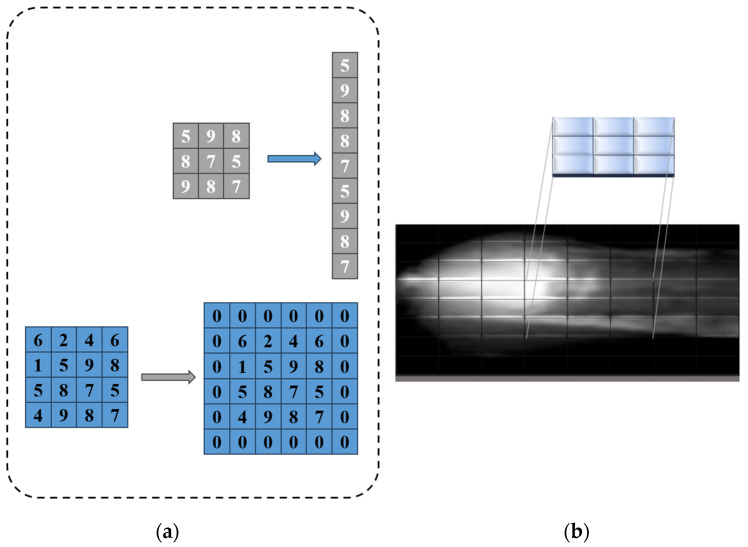
(**a**) Flattening operation & padding operation (**b**) Convolutions dynamics within CNN.

To mitigate computational complexity and enhance runtime speed, images are initially cropped to 880 × 680 pixels and subsequently resized to 256 × 256 pixels to conform to the network’s input requirements. Following this, the K-SVD image enhancement technique mentioned earlier is applied to the images. Additionally, the input images are normalized using mean and standard deviation values of [0.485, 0.456, 0.406] and [0.229, 0.224, 0.225], respectively. Finally, the image inputs are flattened into vectors, as depicted in Figure 7a.

The Figure 7 illustrates the matrix operations: the gray matrix is flattened into column vectors, the blue matrix is expanded by padding zeros, and the arrows indicate the transformation process. Upon entering the network, the images undergo initial feature extraction processes facilitated by convolutional layers. These layers are engineered to reduce the spatial dimensions of the images while simultaneously augmenting the quantity of output channels within the feature maps. The process commences with a 7 × 7 convolutional layer comprising 64 filters, followed by a pooling layer. This sequence persists across subsequent convolutional layers, each varying in the number of filters and pooling layers employed. Following the final convolutional layer, an average pooling layer is introduced. The dynamic movement of kernels over the input image is illustrated in Figure 7b. Each resultant element is derived from the summation of the products obtained by multiplying the feature map with the kernel values at distinct positions. Subsequently, the output of this layer is flattened into a vector and directed into a fully connected layer housing 512 nodes. Ultimately, the output is channeled through another fully connected layer featuring either 6 or 2 nodes.

During the training phase, the objective is to extract adequate feature representations and determine the probability distribution characterizing the dataset. This distribution is defined by weights, encompassing kernels and fully connected layers within the architecture. The training process employs the adaptive moment estimation algorithm for achieving convergence. Initially, a learning rate of 0.001 is utilized to prevent convergence to local optima. Subsequently, the learning rate diminishes by a factor of 0.1 every 10 epochs to ensure optimal convergence. A batch size of 20 samples and a momentum of β = 0.92 are employed during training.

**Figure 8:**
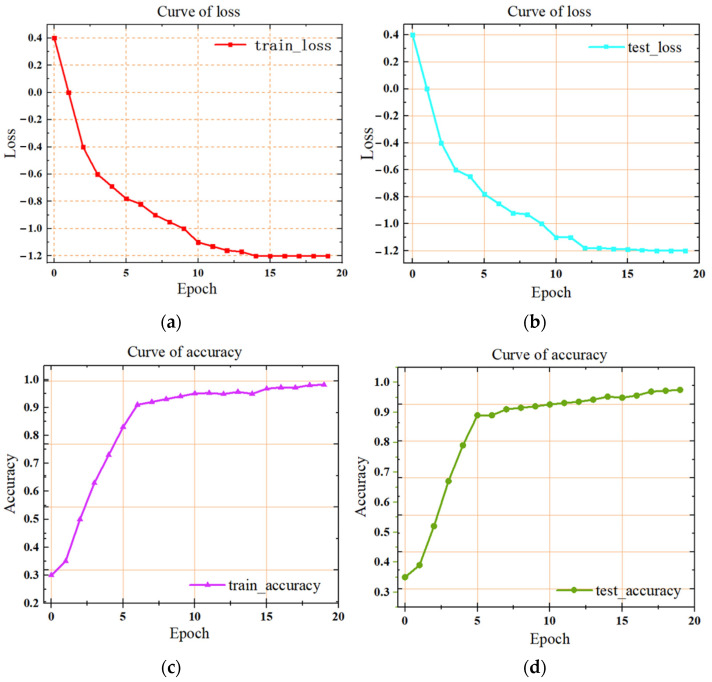
(**a**) Loss curve for the training set (**b**) Loss curve for the test set (**c**) Accuracy curve for the training set (**d**) Accuracy curve for the test set.

In Figure 8, two lines are depicted in red and blue on a graph, with the horizontal axis representing epochs and the vertical axis representing loss. The red training loss curve gradually decreases as epochs progress, while the blue test loss curve also shows a declining trend. To aid visualization, the vertical axis values are plotted using a logarithmic scale with a base of 10. Analysis of the figure reveals that the training loss notably decreases around the 10th epoch, reaching its minimum at approximately the 15th epoch with a value of around −0.8. Similarly, the test loss begins to decrease around the 10th epoch and reaches its lowest point at approximately the 15th epoch. The accuracy of the training set reaches 98.1%, while the accuracy of the test set approaches 98%.

The confusion matrix based on the central loss ResNet in the cross-validation set is provided, where each element in the rows represents the true class, and the elements in the columns represent the predicted class. Larger values on the diagonal indicate better classification performance. From the confusion matrix, it is observed that the ResNet model demonstrates high classification accuracy for welding images. The classification accuracies for good weld bead, burn through, partial-penetration, and undercut are 1.00, 0.960, 0.990, and 0.990, respectively. This result validates the potential application of deep learning in actual K-TIG welding production.

**Figure 9:**
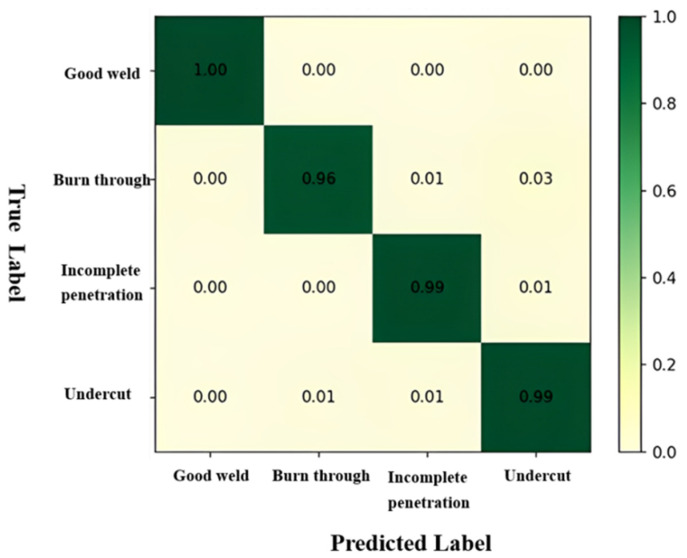
Confusion matrix.

However, from Figure 9, it can be observed that the classification accuracy for burn through is relatively lower compared to the partial-penetration and undercut categories, indicating a mismatch between them. The reason for this phenomenon is the significant overlap observed in the feature space between the burn through category and the partial-penetration and undercut categories. This overlap makes it challenging for the model to accurately distinguish between these categories. The similarity in features among these categories leads to confusion during classification.

The confusion matrix in Figure 9 provides an intuitive reflection of the *F*1 score, precision, and recall for the four categories in TIG welding. The *F*1 score, as defined in Equation (4), combines precision and recall, balancing the performance of the model between positive and negative instances. Precision indicates the proportion of truly defective images among the predicted defective images by the classifier, as shown in Equations (5) and (6), which is crucial for accurate defect detection on production lines. Recall indicates how many truly defective images the model can identify among all defective images. Ensuring that defects are not missed in the TIG welding production environment is vital.

(4) $$Precision=\frac{TruePositive}{TruePositive+FalsePositive}$$

(5) $$Recall=\frac{TruePositive}{TruePositive+FalseNegative}$$

(6) $$F1Score=\frac{2\times (Precision\times Recall)}{Precision+Recall}$$

### 3.2. Comparison with SVM Algorithm

The SVM algorithm can effectively handle data in high-dimensional space [28], which is advantageous considering that images in the K-TIG welding process typically contain a large number of features and dimensions [29,30]. SVM can map the data to a higher-dimensional space by selecting an appropriate kernel function, thereby improving classification and recognition performance.

To compare the performance, the same training and testing datasets are utilized. The primary parameters of SVM include the penalty term C and the kernel parameter γ. In this study, a Gaussian kernel function was employed, with the penalty term C set to 0.01 and the kernel parameter γ set to 1 × 10^−7^. The accuracy and precision of the central loss-ResNet and SVM are compared to assess their performance. The penalty term C = 0.01 and kernel parameter γ = 1 × 10^−7^ were chosen for the SVM algorithm based on their effectiveness in balancing the trade-off between margin maximization and error minimization, as well as their ability to handle high-dimensional data and improve classification performance.

**Figure 10:**
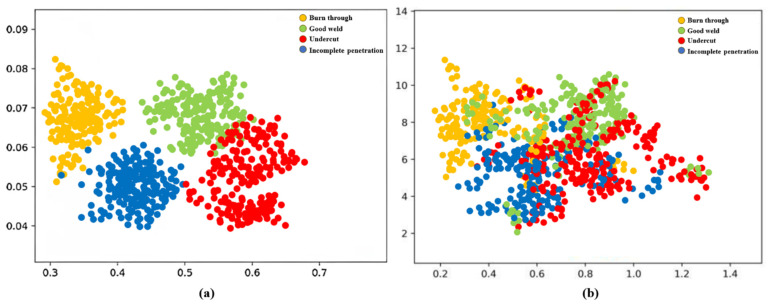
Visualization of deep feature: (**a**) Combination of softmax loss and center loss (**b**) Softmax loss only.

From Table 3, it is evident that while SVM can achieve relatively high precision in identifying underpenetrated, good welds, and bite edges, it exhibits poor accuracy in identifying burn-through images. On the other hand, the central loss ResNet achieves 100% accuracy in identifying burn-through images. This highlights the superiority of the central loss ResNet in welding image classification. The classification effectiveness is further illustrated in Figure 10. The horizontal and vertical coordinates represent the coordinates in the feature space, and usually do not need a specific physical meaning, and the representative is the distribution map in the feature space.

### 3.3. Visual Explanation

Interpretability is a crucial concern within the realm of deep learning [31], particularly in important application domains like the identification of defects in welding procedures [32]. Deep learning models are commonly perceived as opaque constructs, with their internal mechanisms proving challenging to elucidate and comprehend. Nevertheless, in critical sectors such as welding quality assurance, interpretability plays a pivotal role in facilitating decision-making processes and validation procedures [33,34]. The Integrated Gradients algorithm represents a technique designed to elucidate the predictions made by a model by scrutinizing the model’s responsiveness to input features [35], thereby shedding light on the model’s decision-making rationale. This algorithm operates on the foundational principles of sensitivity and implementation invariance [36], with the objective of furnishing an assessment of the impact of input features on alterations in the model’s output, essentially delineating the significance of these features [37,38].

Leveraging class-specific gradient data from the ultimate convolutional layer of a convolutional neural network, the Integrated Gradients algorithm can generate rudimentary maps pinpointing crucial regions within an image. Referring to the ResNet model as *F*, which maps the input tensor x to the output *F(x)*, a specific baseline point *x*’ is designated, typically representing the image in its pure black form. This baseline point is intricately linked to the input *x*, yet exerts minimal influence on the model’s predictive outcomes. The Integrated Gradients, denoted as G, quantify the cumulative impact of each feature xi on alterations in the model’s output, as delineated in Equation (7). At each interpolation juncture, the partial derivative of the model’s output concerning feature xi is computed, and these gradient values are aggregated across feature *i* within the interval spanning from α = 0 to 1. The cumulative gradients are subsequently multiplied by (*x_i_* − *x_i_*′) to yield the ultimate integrated gradient.

(7) $$IG_{i}(x)=(x_{i}-x_{i}^{\prime })\int_{a=0}^{1}\frac{\partial F(x^{\prime }+a(x-x^{\prime }))}{\partial x_{i}}d\alpha$$

**Figure 11:**
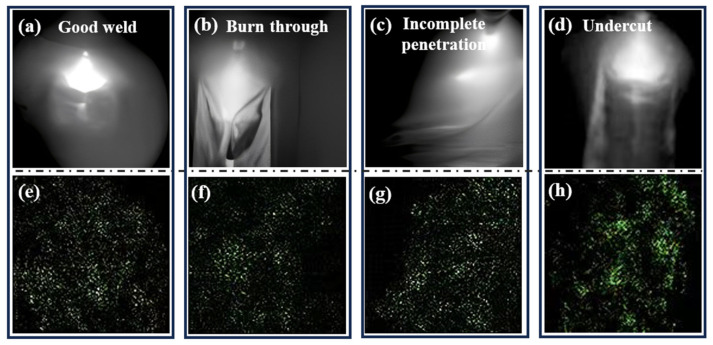
Example of an image processed by the algorithm (**a**–**d**) Input Image; (**e**–**h**) After Integrated Gradients.

The illustration in Figure 11 demonstrates a clear emphasis on the pore and the adjacent molten pool area, suggesting that significant information has been derived from this specific region. The green sections in the image correspond to edges or outlines, accentuating intricate details. Notably, the texture of the molten pool is identified as the key characteristic for the classification of “partial penetration”. In this context, the pertinent features distributed across the partial penetration zones are leveraged to ascertain their classification. Consequently, it is posited that ResNet exhibits proficiency in capturing distinctive features relevant to class differentiation.

## 4. Conclusions

This research introduces a novel monitoring system for observing the welding pool and its vicinity using images obtained from an HDR camera during TIG welding, complemented by a processing approach based on ResNet. Unlike prior investigations, this study emphasizes spectral analysis for identifying welding imperfections and integrates laser lighting to address the inherent uncertainty in attaining optimal weld integrity. The HDR camera effectively eliminates strong arc light emissions, ensuring image equilibrium and exposing details of the solder pool. Utilizing ResNet’s capabilities, specific defects in welds can be accurately pinpointed by discerning critical differences between flawless and flawed welds. A comparative evaluation of defect classification performance between ResNet and SVM illustrates ResNet’s superior ability to detect more intricate defects while maintaining a favorable balance between correctly identifying one defect and misclassifying another, resulting in an overall accuracy rate of 98.475%. These results underscore ResNet’s potential to effectively adapt to industrial demands, boost efficiency, and consistently achieve superior TIG welding results.

The significance of these findings lies in their potential practical implications for industrial applications. The integration of ResNet with HDR imaging offers a robust solution for real-time weld monitoring and defect detection, addressing the challenges of variability in welding conditions. This method can significantly enhance production efficiency and quality control in various industrial settings.

However, the training and deployment of deep neural networks require significant computational resources, which may not be feasible for all industrial environments. The study primarily focuses on keyhole TIG welding, necessitating further investigation to apply this method to other welding techniques. Future research could integrate additional sensor data, such as thermal or acoustic signals, to enhance defect detection accuracy and reliability. Additionally, developing interfaces and tools for human-AI collaboration in welding inspection could combine human expertise with artificial intelligence for better results, thus broadening the applicability and impact of this research in the field of industrial welding.

## Figures and Tables

**Table 1:** TIG welding experimental instrument parameters.

Welding Current (A)	Welding Speed (cm/min)	Type
450–460	35	Good weld
500–550	35	Burn through
400–410	35	Undercut
330–350	35	Partial-penetration

**Table 2:** Dataset split between training, test and validation.

Category	Training Dataset	Test Dataset	Validation Dataset
good weld	1200	290	350
defect	1200	510	450
Total	2400	800	800

**Table 3:** Comparison of algorithms.

Algorithm	Center Loss-Resnet		SVM	
Evaluation index	Precision	Accuracy	Precision	Accuracy
Burn through	1.000	1.000	1.000	0.295
Good weld	0.975	0.948	0.565	0.914
Undercut	0.968	0.981	0.965	0.985
Partial-penetration	0.996	0.997	0.997	0.997

## Data Availability

The data presented in this study are available on request from the corresponding authors.
